# Supplementary material for: Alienation in Ethiopian cinema: “T’eza” (“Morning Dew”) and “Səlä ʾänəči” (“About you”) in focus
Source: PLoS One. 2025 Apr 15;20(4):e0317311. doi: 10.1371/journal.pone.0317311 (PMC11999118; doi:10.1371/journal.pone.0317311)
Supplement: Attachment — Submitted filename: Response to Reviewers.docx [file pone.0317311.s003.docx]

**'Response to Reviewers'**

We sincerely thank you for your valuable feedback and constructive comments on our manuscript, Alienation in Ethiopian Cinema: *'T'eza' ('Morning Dew')* and *'Səlä ʾänəči' ('About You')* in Focus. We deeply appreciate the time and effort invested in reviewing our work and are grateful for the opportunity to address the suggestions and resubmit a revised version.

We have carefully addressed each point raised by the reviewers and have made the necessary revisions to improve the clarity and depth of the manuscript. In the revised manuscript, we have incorporated feedback from the academic editor and reviewers. To highlight the changes made in response to their comments, we have used **green colour to indicate addressing the academic editor’s suggestions taken from reviewers’ comments** and red colour to indicate addressing Reviewer's comments.

This allows for easy tracking of the revisions made in response to each feedback.

**Academic Editor Comments Taken from Reviewers**

Your insightful comment was invaluable. We've incorporated it into a revised manuscript that reflects the valuable feedback.

| Academic Editor’s Comment based on the reviewers’ suggestions | Authors’ Response | Location |
| --- | --- | --- |
| - The reviewers requested you to clarify some points, give a short contextualization of the Derg regime, if possible, relate your research to the previously published literature, and verify your reference list. | - Thank you for your valuable feedback on our manuscript. In response to your comments, we have clarified key points, provided a concise contextualization of the Derg regime by outlining its Marxist-Leninist ideology, repressive governance, and the resulting societal alienation reflected in the films. Our study examines alienation in Amharic films through psychoanalytic and Marxist lenses, focusing on the socio-political conditions of the Derg regime (1974–1991). This era, marked by authoritarianism, civil war, and disillusionment, fostered deep psychological and ideological alienation, particularly among intellectuals and artists. Building on works like [3], we analyse characters struggling with trauma, dislocation, and ideological crises shaped by the regime’s oppressive policies. - Unlike [39] global critique of capitalism in Ethiopian cinema, our study emphasizes localized struggles under the Derg, highlighting the emotional dimensions of alienation. Revisions clarify the focus on psychological and ideological disillusionment, integrating Freudian and Marxist analyses of characters like Anberber in *T'eza*. This approach deepens understanding of alienation within Ethiopia’s unique political history. | - Theoretical Framework Section - Analysis Section |
| - Please review your reference list to ensure that it is complete and correct. If you have cited papers that have been retracted, please include the rationale for doing so in the manuscript text, or remove these references and replace them with relevant current references. | - We sincerely appreciate your careful review and valuable feedback on our reference list. Following your suggestions, we have conducted a comprehensive review to ensure all citations are complete, accurate, and conform to the journal referencing style. For any retracted papers cited, we have either provided a clear rationale for their inclusion within the manuscript or noted their retracted status in the references section, accompanied by the full citation of the retraction notice. In cases where replacement was appropriate, we have substituted them with current and relevant sources. | - Reference Section |

**Reviewer # 1**

- Reviewer # 1 Since the first reviewer did not provide any comment I try to address his/her comment in line with the comments forwarded by the editor. Therefore, no response is required.

**Reviewer # 2**

Your insightful comments were invaluable. We've incorporated them into a revised manuscript that reflects the valuable feedback.

| Reviewers’ Comment | Authors’ response | Location |
| --- | --- | --- |
| - I think the later sections of the essay could better dialogue with the work of their colleagues Bitania Tadesse and Steven Thomas, whom they mention only at the beginning, but whose work relates to theirs significantly. Therefore, the authors might want to consider that conversation among scholars of Ethiopian cinema as being slightly more interesting to readers than the survey of old theory. - Moreover, considering *T’eza’s* focus on the Derg regime, I wonder why there is so little information about Ethiopia's political and economic history. Nevertheless, the essay stands and does what it purports to do, which is read the films through the lenses of some Marxist and Freudian theories. | - Thank you for your thoughtful and comprehensive feedback. - Our study benefits from engaging with the work of [11] and [39], whose research provides essential context for Ethiopian cinema. Bitania’s analysis of socio-political and historical narratives in films like *T'eza* and *Səlä ʾänəči* offers valuable background on class struggles and ideological conflicts, but it does not address psychological and existential alienation. This gap is filled by our psychoanalytic and Marxist approach, which explores how external societal forces shape individual identity and perception. - [39] Marxist critique, drawing on Fredric Jameson, highlights systemic economic and cultural alienation in films such as *Rebuni* and *YeWendoch Guday*, but does not explore personal, psychological experiences. Our study, by focusing on trauma and identity in *T'eza* and *Səlä ʾänəči*, examines how these macro-level themes translate to individual alienation. - By integrating historical and cultural context with psychoanalytic insights, we enrich the discourse on Ethiopian cinema. [11] work complements our focus by connecting transnational and cultural alienation, while [39] theories deepen the discussion of class dynamics. A more thorough comparative dialogue with these scholars situates our research within ongoing Ethiopian film studies and offers a more compelling exploration of localized psychological alienation. - In the film *T'eza*, we added an extract in political alienation that is powerfully conveyed through Anberber’s critique of the endless cycles of war. In *T'eza*, Anberber’s critique of the endless war underscores political alienation, as his frustration with the government's and opposition's focus on power over the people's welfare aligns with Marx's theory of estrangement from oppressive systems. The warring factions exemplify class oppression, using youth as expendable resources, echoing Gramsci's theory of hegemonic power and normalization of conflict. [6] idea of ideological state apparatuses reveals how war serves as a tool for ideological control, fostering despair. Anberber’s disillusionment mirrors [34] notion of political alienation, where citizens withdraw from a corrupt system. - In *T'eza*, Anberber’s encounter with Worku’s mother, carrying her wounded son and his military boots—a symbol of meager compensation—highlights economic alienation. Worku’s sacrifices and service are undervalued, exposing the exploitation and inequality inherent in the system. Marx's theory of alienation is evident as Worku’s labor is commodified, and [6] concept of ideological state apparatuses shows how the military perpetuates acceptance of such systemic inequality, disguising it as duty. [22] cultural hegemony reveals that cultural narratives normalize this exploitation. Worku’s suffering and his mother’s burden underscores the broader alienation of the working class. Anberber’s awareness of Worku’s plight shifts his perspective from personal grievances to a critique of class-based injustice. | - Theoretical framework - Analysis Section |
| - The only absolutely necessary change is the references page, where some of the references are incomplete or improperly formatted according to the style guidelines. That is a problem, but it is a problem that can be easily and quickly resolved. | - Thank you for your thorough review and valuable feedback on our manuscript. - We appreciate your attention to detail, particularly concerning the formatting of the references section. We acknowledge that some references were incomplete or did not adhere to the style guidelines. We understand the importance of precise formatting and have taken immediate steps to rectify this issue. - In response, we have carefully reviewed and revised the entire references section to ensure that all entries now meet referencing requirements. While this adjustment required careful attention, we believe it does not impact the scholarly quality, originality, or strength of the manuscript's arguments and analyses. This issue was limited to the formatting of the references page and has been promptly resolved. We trust that this correction meets the high standards expected and appreciate your understanding and constructive guidance. | - Reference Section |
